# Properties of Silicone Rubber-Based Composites Reinforced with Few-Layer Graphene and Iron Oxide or Titanium Dioxide

**DOI:** 10.3390/polym13101550

**Published:** 2021-05-12

**Authors:** Vineet Kumar, Anuj Kumar, Minseok Song, Dong-Joo Lee, Sung-Soo Han, Sang-Shin Park

**Affiliations:** 1 School of Mechanical Engineering, Yeungnam University, 280 Daehak-ro, Gyeongsan 38541, Korea; vineetfri@gmail.com (V.K.); djlee@yu.ac.kr (D.-J.L.); 2 School of Chemical Engineering, Yeungnam University, 280 Daehak-ro, Gyeongsan 38541, Korea; anuj.budhera@gmail.com (A.K.); sshan@yu.ac.kr (S.-S.H.); 3 Graduate School of Mechanical Engineering, Yeungnam University, 280 Daehak-ro, Gyeongsan 38541, Korea; masonsong616@gmail.com

**Keywords:** rubber composites, hybrid filler, few-layer graphene, titanium dioxide, iron oxide

## Abstract

The increasing demand for polymer composites with novel or improved properties requires novel fillers. To meet the challenges posed, nanofillers such as graphene, carbon nanotubes, and titanium dioxide (TiO_2_) have been used. In the present work, few-layer graphene (FLG) and iron oxide (Fe_3_O_4_) or TiO_2_ were used as fillers in a room-temperature-vulcanized (RTV) silicone rubber (SR) matrix. Composites were prepared by mixing RTV-SR with nanofillers and then kept for vulcanization at room temperature for 24 h. The RTV-SR composites obtained were characterized with respect to their mechanical, actuation, and magnetic properties. Fourier-transform infrared spectroscopy (FTIR) analysis was performed to investigate the composite raw materials and finished composites, and X-ray photoelectron spectroscopy (XPS) analysis was used to study composite surface elemental compositions. Results showed that mechanical properties were improved by adding fillers, and actuation displacements were dependent on the type of nanofiller used and the applied voltage. Magnetic stress-relaxation also increased with filler amount and stress-relaxation rates decreased when a magnetic field was applied parallel to the deformation axes. Thus, this study showed that the inclusion of iron oxide (Fe_3_O_4_) or TiO_2_ fillers in RTV-SR improves mechanical, actuation, and magnetic properties.

## 1. Introduction

Silicone rubber (SR) is frequently used as a composite matrix because it is easily made and has high dielectric and mechanical properties [1,2]. SR is used in a variety of applications such as in actuators [2], strain sensors [3], and coatings [4,5,6]. Among its industrial applications, SR is commonly used to produce actuators and strain sensors [2,3]. With regards to fillers, nanofillers such as carbon nanotubes (CNTs) [7], graphene, few-layer graphene (FLG) [8], nano-titanium dioxide (TiO_2_) [9], and iron oxide (Fe_3_O_4_) [10] are often used to produce composites. FLG is used for its high mechanical properties [11,12], TiO_2_ for its high dielectric constant [13,14], and Fe_3_O_4_ for its ability to orient in a magnetic field and act as a magnetic sensor [15]. From the composite point of view, various types of nanofillers and polymer matrices are mixed to obtain high-performance polymer composites [16] and, in this context, composites based on SR and nanofillers such as CNTs [17], graphene [18], few-layer graphene [19], and carbon black [20,21] are frequently used.

The main role of fillers in rubber composites is to improve mechanical and electrical properties. Several ways have been devised to improve composite properties, for example, (a) using modified (modified or functionalized) fillers [22,23] or polymer matrices to improve interfacial interactions; (b) using hybrid (hybrid or more than one) fillers that exhibit synergistic effects in composites [24]; (c) using different types of polymers (e.g., elastomers [25], thermoplasts [26], and thermosets [27]) to improve composite moduli; and (d) using polymer blends [28]. Of these possibilities, hybrid fillers that exhibit synergistic effects [29] or improve interfacial interaction [30] are usually used. Recent research also showed results on oriental lacquers using atomic force microscopy [31,32].

Hybrid fillers are critical in improving the reinforcing and fatigue properties of rubber composites [33]. The use of fillers in a hybrid form exhibit the additional improvement of reinforcing properties due to the synergistic effect [34]. The use of FLG in hybrid with iron particles not only exhibit higher mechanical properties, but also act as a magnetic sensor to be used for applications such as magneto-rheological effects (MREs). MREs are a class of smart materials where the reinforcing properties can be controlled by an external magnetic field [35]. MREs consists of a rubber or polymer matrix reinforced with iron particles, which can orient in the direction of a magnetic field [35]. Depending on the subjecting of the magnetic field, they can be categorised as an isotropic and anisotropic class of MREs [36]. The electro-active, but stretchable electrode in actuation requires continuous external voltage to produce displacements and mechanical motions for thin and flexible devices. Graphitic-based materials acting as an electrode can be useful for diverse chemical properties and can act as both an oxidizing and reducing agent in the electrode used for actuation purposes [37]. Few studies have investigated the use of FLG in polymer composites [38], probably because of its cost [39,40].

However, studies showed that the use of FLG as a filler improves mechanical and electrical properties [38]. On the other hand, many studies have investigated the photocatalytic [41] and anti-corrosive [42] effects of nano-TiO_2_ in polymer matrices, and a few have explored the MREs of Fe_3_O_4_ in elastomers [43]. However, very few studies have addressed the use of RTV-SR containing FLG and TiO_2_ or Fe_3_O_4_ for actuators or magnetic sensors as end uses. We provide the effects of nanofillers on the mechanical properties of composites. In addition, we examined the effects of fillers on actuator and magnetic sensor properties. The target application of this work is soft materials for actuation and MREs. Moreover, FLG was used as a secondary reinforcing filler in hybrid RTV-SR/FLG-Fe_3_O_4_, while Fe_3_O_4_ was used as a magnetic sensing agent for MREs.

## 2. Materials and Methods

### 2.1. Materials

RTV silicone rubber (SR) (KE-441, Shin-Etsu, Japan) was used as the polymer matrix for preparing SR composites, and CAT-RM (Shin-Etsu, Japan) was used as the vulcanizing agent. The fillers used to reinforce the SR matrix were Fe_3_O_4_ (US Research Nanomaterials, Inc., Houston, TX, USA), FLG (Asbury Mills, Asbury, NJ, USA), and TiO_2_ (Alfa Aeser, Ward Hill, MA, USA). The mould release agent was purchased from Nabakem.

### 2.2. Fabrication of Nanocomposites

**Figure 1:**
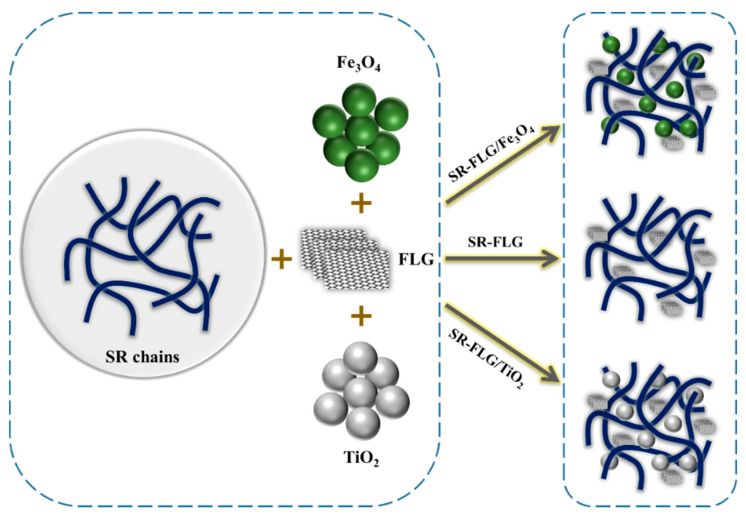
A schematic illustration of the preparation of rubber-based composites.

Composite preparation at room temperature was started by spraying a mould with the mould release agent and leaving it to dry for 3 h. The RTV-SR was placed in a container and mixed manually with a known amount of filler for 10 min. Then, 2 phr vulcanizing agent was added and mixed; the mixture was then poured into a mould and left for 24 h at room temperature to cure. The composites obtained were then removed from the moulds and tested. Samples were designated RTV-SR/FLG, RTV-SR/FLG-Fe_3_O_4_, and RTV-SR/FLG-TiO_2_ as described in Table 1. A schematic illustration of the preparation of the RTV-SR composites is shown in Figure 1.

### 2.3. Characterisation

#### 2.3.1. SEM Micrographs

Composite and filler powder morphologies and surface compositions were studied by SEM (S-4100, Hitachi, Tokyo, Japan) and EDX, respectively.

#### 2.3.2. XRD Analysis

Crystal structures were studied by XRD (D8, Advance Bruker) at room temperature (25 °C) and a scan rate of 10° min^−1^.

#### 2.3.3. BET Analysis

Adsorption isotherms were obtained using a BELSORP-max (BEL, Osaka, Japan) at 77 K to estimate BET surface areas of fillers.

#### 2.3.4. Elemental Mapping through SEM

The elemental mapping and morphologies were studied by SEM (s-4100, Hitachi). RTV-SR composites with 20 phr filler were sectioned using a surgical blade to 0.5 mm thick. Samples were also ion-sputter coated with platinum for 2 min. In addition, the presence of Si, O, Fe, C, and Ti elements and their distributions in RTV-SR composites were investigated by X-ray mapping (Horiba EMAX, Tokyo, Japan).

#### 2.3.5. FTIR Analysis

FTIR (Perkin Elmer) was used to identify functional groups of fillers and RTV-SR composites in the transmittance mode over the range 4000–600 cm^−1^.

#### 2.3.6. XPS Analysis

XPS (ESCALAB 250), with a monochromatic Al K_σ_ X-ray source (hν = 1486.6 eV; spot size 200-μm), was used to determine the binding energies of interactions in RTV-SR composites.

#### 2.3.7. Mechanical Properties

Mechanical properties were studied using a universal testing machine (UTM, Lloyd Instruments, West Sussex, UK) at a strain rate of 2 mm/min using cylindrical samples (diameter 20 mm and height 10 mm) at a strain rate of 200 mm/min using dumble-shaped specimens (2 mm thick and with a gauge length of 25 mm).

#### 2.3.8. Hardness

Shore a hardness of the rubber composites were measured using a Westop durometer. A Shore A hardness test is a fast, easy and suitable method for measuring the hardness of a rubber composite. The operation included the application of manual force to the sample and adjusting the testing height to obtain measurements for the specimen with a thickness of around 10 mm.

#### 2.3.9. Actuation Measurements

Actuation displacements were measured using a laser sensor (OptoNCDT 1302) using a 0.1 mm thick electrode with a diameter of 25 mm. The elastomer slab, on the other hand, was 1 mm thick and made of 3 M silicone rubber. The loading of the electrode was 15 phr of nanofiller (FLG, FLG-Fe_3_O_4_, or FLG-TiO_2_) in RTV-SR.

#### 2.3.10. Magnetic Stress-Relaxation Tests

Stress-relaxation Measurements were obtained in the presence of a magnetic field using a universal testing machine (UTM, Lloyd Instruments, West Sussex, UK) using the same cylindrical samples used for mechanical testing.

## 3. Results

### 3.1. Morphology and Purity of Filler Particles

**Figure 2:**
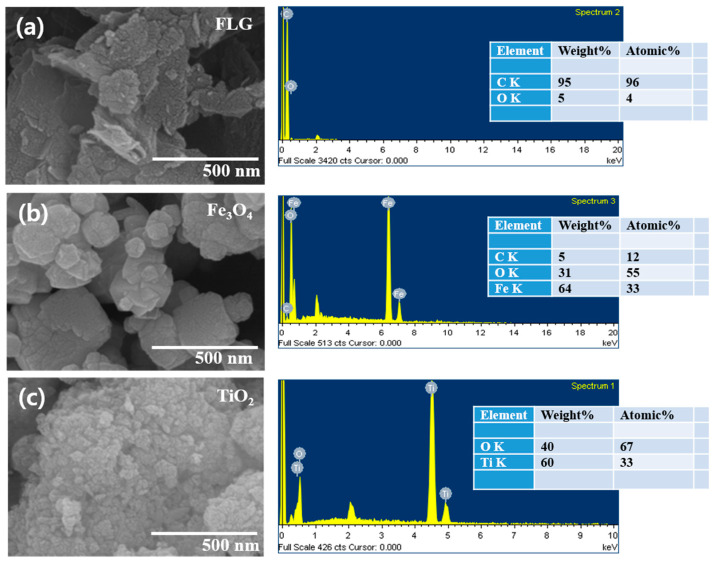
SEM micrographs and EDX spectra of (**a**) FLG, (**b**) Fe_3_O_4_, and (**c**) TiO_2_.

The microstructures of the nanofillers were studied by SEM. FLG had a sheet-like morphology with an ordered distribution of the sheets and a thickness of up to 9–12 nm. A SEM of Fe_3_O_4_ revealed the particles were cubic and had a random distribution, whereas TiO_2_ particles were spherical with random distribution of the particles and sizes in the range of 17–21 nm. The purities of filler particles are known to influence composite properties. Kumar et al. demonstrated the importance of filler purity in their studies based on SWCNT reinforced rubber composites [44]. We determined the chemical purities of fillers by SEM and EDX (Figure 2a (FLG), Figure 2b (Fe_3_O_4_), and Figure 2c (TiO_2_)). Results showed the purities of FLG, Fe_3_O_4_, and TiO_2_ were 95%, 95%, and 99%, respectively.

### 3.2. X-ray Diffraction and Adsorption Isotherms of the Fillers

**Figure 3:**
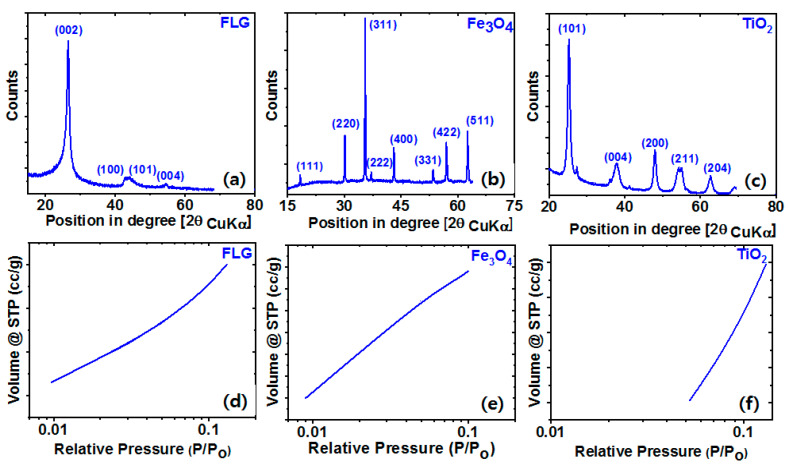
XRD spectra and adsorption isotherms of (**a**,**d**) FLG, (**b**,**e**) Fe_3_O_4_, and (**c**,**f**) TiO_2_.

The crystalline states of FLG, Fe_3_O_4_, and TiO_2_ were determined by XRD (Figure 3a–c). A distinct (002) peak at around 2θ = 25.34° was observed in the XRD spectrum of FLG indicating a crystalline state. This peak can be used to calculate the number of graphene layers stacked in crystalline domains. XRD showed Fe_3_O_4_ and TiO_2_ had highly crystalline structures. Characteristic 2θ peaks for Fe_3_O_4_ were observed at 18.3°, 30.1°, 35.5°, 37.1°, 43.1°, 53.4°, 57.1°, and 62.6°, which corresponded to the (111), (220), (311), (222), (400), (331), (422), (511) planes of crystal type Fe_3_O_4_ [45]; and characteristic peaks for TiO_2_ were observed at 25.3°, 37.8°, 48.1°, 55.1°, and 62.5°, which corresponded to (101), (004), (200), (211), and (204) [46]. Furthermore, XRD showed no evidence of an amorphous phase in Fe_3_O_4_, FLG, or TiO_2_. The filler surface properties were determined using adsorption isotherms (Figure 3d–f) and showed volumes of adsorbed gas were related to partial pressure. At a partial pressure of 0.1, BET surface areas for FLG, Fe_3_O_4_, and TiO_2_ were 410 m^2^/g, 5 m^2^/g, and 180 m^2^/g, respectively.

### 3.3. Filler Dispersions by Elemental Mapping in Nanocomposites

**Figure 4:**
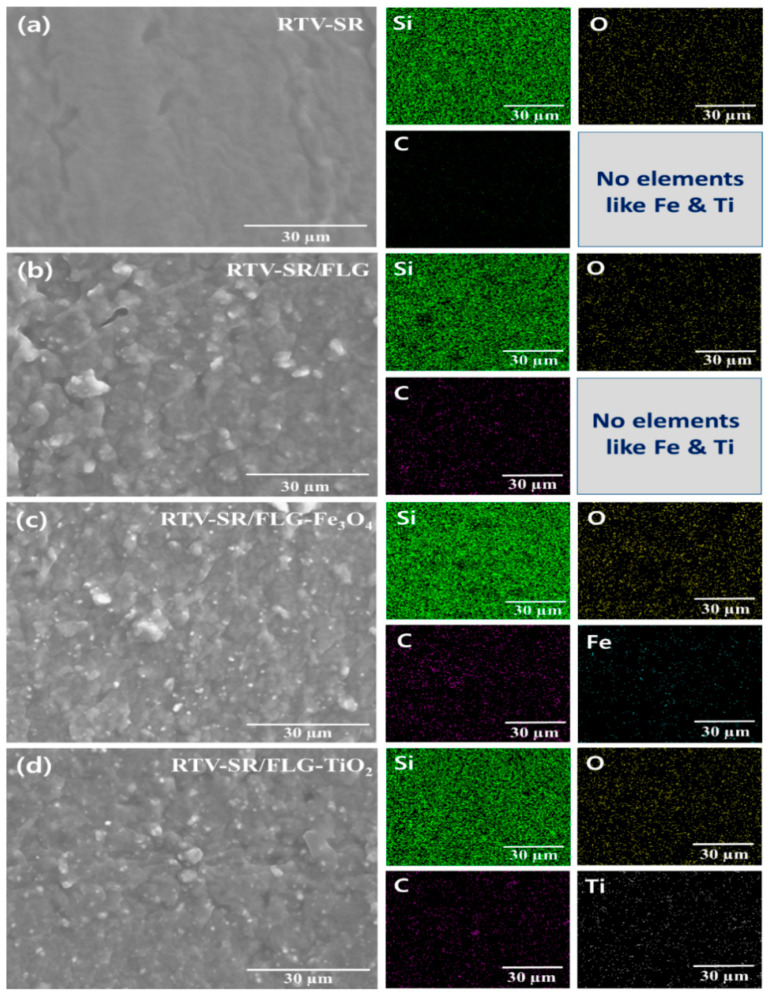
Filler distributions as determined by elemental mapping: (**a**) RTV-SR, (**b**) RTV-SR/FLG, (**c**) RTV-SR/FLG-Fe_3_O_4_, and (**d**) RTV-SR/FLG-TiO_2_.

The Filler dispersions at a filler loading of 20 phr were determined by elemental mapping (Figure 4). Figure S1 showed that the filler particles were dispersed uniformly in the RTV-SR. Filler particles are indicated by arrows in the high-resolution SEM image.

The homogenous dispersions of nanofillers were confirmed by EDX (Figure 4). Si, O, and C were densely distributed in all composites (Figure 4a–d). Carbon, originated from FLG and RTV-SR (Figure 4a–d), was well distributed and showed the formation of percolative networks. However, some aggregate formation was observed in C maps, indicating some reduction in mechanical properties. The Fe map (Figure 4c) revealed a random distribution of Fe_3_O_4_ particles and no evidence of percolative networks of Fe_3_O_4_ particles. Similarly, the Ti map (Figure 4d), which represented the distribution of TiO_2_, showed a random distribution without percolative network formation or signs of aggregation.

### 3.4. FTIR Analysis

**Figure 5:**
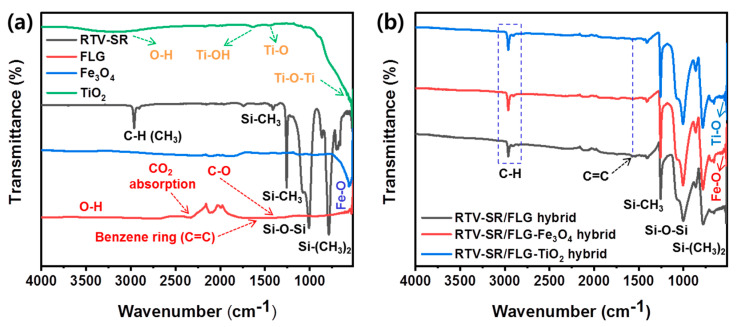
The (**a**) FTIR spectra of RTV-SR, FLG, Fe_3_O_4_, and TiO_2_, and (**b**) the three composites (RTV-SR/FLG, RTV-SR/FLG-Fe_3_O_4_, and RTV-SR/FLG-TiO_2_).

FTIR analysis was performed on a powder sample and the composites based on a 20 phr filler loading and SR matrix. FTIR was used to study the functional groups in the composites (Figure 5). RTV-SR exhibited characteristic peaks at ~2961 cm^−1^ (C-H stretch in CH_3_), 1413 and 1252 cm^−1^ (CH_3_ deformation mode), 1082 and 1015 cm^−1^ (Si-O-Si stretch), and 791 cm^−1^ (Si-C stretch in Si-(CH_3_)_2_) [47]. Major featured peaks of FLG were observed at ~1628 and ~1472 cm^−1^ (assigned to aromatic C=C) [48]. Additionally, characteristic peaks were observed at ~2320 cm^−1^ (due to absorbed environmental CO_2_), 1084 cm^−1^ (C-OH), and 1085 cm^−1^ (C-O) [48,49]. TiO_2_ produced major peaks at ~3497 cm^−1^ (broad peak, O-H stretch), 1630 cm^−1^ (-OH bending mode of Ti-OH), 1384 cm^−1^ (assigned to Ti-O), and 524 cm^−1^ (broad peak assigned to Ti-O-Ti stretch) [50,51,52]. Fe_3_O_4_ showed a major peak at ~556 cm^−1^ due to Fe-O stretch [53]. However, the introduction of fillers caused no major shift in the characteristic peaks of RTV-SR, whereas the formation of hydrogen bonding between Si-O-Si (from RTV-SR) and oxygen-containing groups from FLG, Fe_3_O_4_, and TiO_2_ as fillers were formed.

### 3.5. XPS Analysis

**Figure 6:**
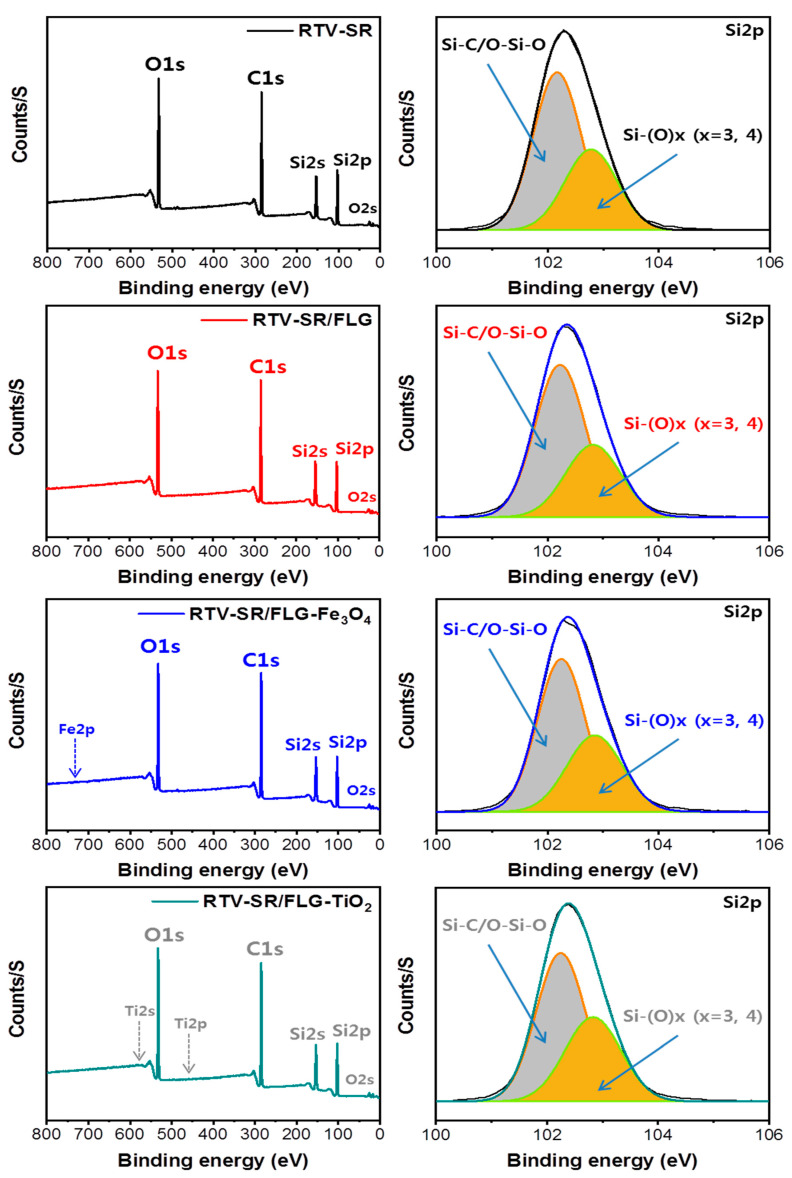
Full survey XPS scans of RTV-SR, RTV-SR/FLG, RTV-SR/FLG-Fe_3_O_4_, and RTV-SR/FLG-TiO_2_, and their corresponding high resolution de-convoluted Si2p XPS spectra.

**Figure 7:**
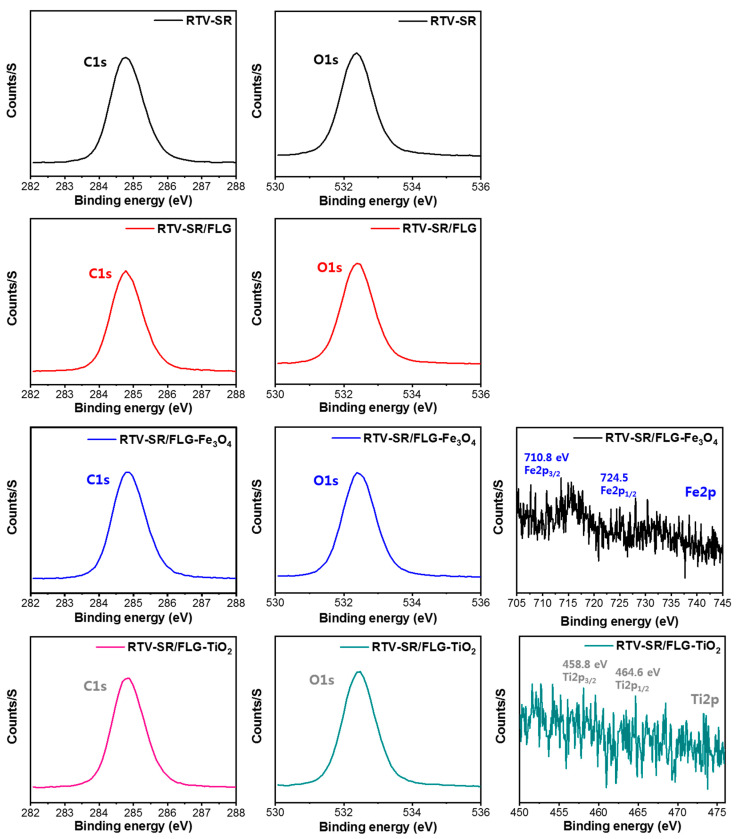
High-resolution XPS of C1s, O1s, Fe2p, and Ti2p spectra of the base polymer (RTV-SR) and the three composites.

The changes in the surface elemental compositions of RTV-SR composites were assessed by XPS (Figure 6 and Figure 7). Figure 6 shows the XPS scans of RTV-SR, RTV-SR/FLG, RTV-SR/FLG-Fe_3_O_4_, and RTV-SR/FLG-TiO_2_, and their corresponding high-resolution XPS Si2p spectra.

Full survey XPS spectra of RTV-SR and RTV-SR/FLG clearly showed the presence of carbon (C1s), oxygen (O1s), and silicone (Si2s and Si2p), while peaks of iron (Fe2p) [54] and titanium (Ti2s and Ti2p) [55] were observed after incorporating Fe3O4 and TiO2 in RTV-SR/FLG composites (Figure 6 and Figure 7). Furthermore, in the high-resolution XPS spectra of RTV-SR, two main peaks of silicone rubber chains were observed at 102.4 eV (Si-C/O-Si-O ascribed to organic silicone) and 103.5 eV (Si-(O)x, x = 3, 4; inorganic silicone), which represented silicone backbones and side groups, respectively [56,57]. However, no significant changes in the heights or shifts of high-resolution XPS Si2p peaks were observed between composites. The high-resolution XPS spectra of C1s, O1s, Fe2p, and Ti2p are provided in Figure 7.

### 3.6. Compressive Mechanical Properties

**Figure 8:**
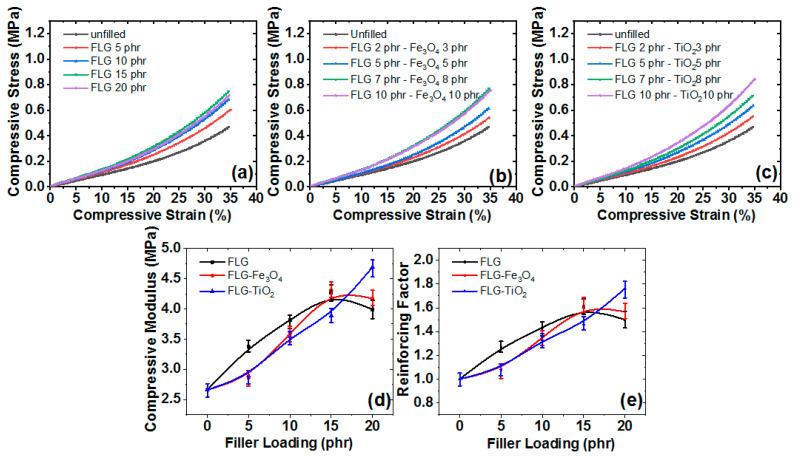
Compressive mechanical properties; (**a**) stress-strain plots of RTV-SR/FLG, (**b**) RTV-SR/FLG-Fe_3_O_4_, (**c**) RTV-SR/FLG-TiO_2_, (**d**) compressive modulus plots, and (**e**) reinforcing factor plots of the three composites.

The compressive properties of the three composites are shown in Figure 8. Compressive stress and strain were evaluated for FLG (Figure 8a), FLG-Fe_3_O_4_ (Figure 8b), and FLG-TiO_2_ composites (Figure 8c). Compressive stress increased with compressive strain. Compressive stress at a filler loading of 15 phr was 0.02 MPa for RTV-SR/FLG, 0.019 MPa for RTV-SR/FLG-Fe_3_O_4_, and 0.016 MPa for RTV-SR/FLG-TiO_2_ at 1% strain, and increased to 0.74 MPa for FLG, 0.76 MPa for FLG-Fe_3_O_4_, 0.73 MPa for FLG-TiO_2_ at 35% strain, which we supposed was due to the increased packing fraction of filler networks on increasing compressive strain. Similarly, compressive stress increased with increasing filler content. Compressive stress at 35% compressive strain and a 5 phr filler loading was 0.6 MPa for RTV-SR/FLG, 0.54 MPa for RTV-SR/FLG-Fe_3_O_4_, 0.55 MPa for RTV-SR/FLG-TiO_2_, and increased to 0.74 MPa for RTV-SR/FLG, 0.76 MPa for RTV-SR/FLG-Fe_3_O_4_, 0.73 MPa for RTV-SR/FLG-TiO_2_ at a loading of 15 phr. On the other hand, compressive stress decreased upon increasing filler loading from 15 to 20 phr for RTV-SR/FLG and RTV-SR/FLG-Fe_3_O_4_, possibly due to filler particle aggregation [58]. Compressive moduli (Figure 8d) and reinforcing factors (Figure 8e) were measured for the three composites. Both variables increased on increasing filler loading up to 15 phr for RTV-SR/FLG and RTV-SR/FLG-Fe_3_O_4_ and up to 20 phr for RTV-SR/FLG-TiO_2_. The compressive modulus at a filler loading of 5 phr was 3.37 MPa for RTV-SR/FLG, 2.86 MPa for RTV-SR/FLG-Fe_3_O_4_, and 2.89 MPa for RTV-SR/FLG-TiO_2_, and these increased at 15 phr to 4.27, 4.31, and 3.89 MPa, respectively. However, compressive moduli fell upon increasing loading from 15 to 20 phr for FLG and the FLG-Fe_3_O_4_ hybrid, which we attributed to the aggregation of filler particles at high filler contents and non-uniform filler dispersions [58]. The better properties of the RTV-SR/FLG-TiO_2_ hybrid at 20 phr are believed to be due to some synergistic effect between filler particles [24,29].

### 3.7. Tensile Mechanical Properties

**Figure 9:**
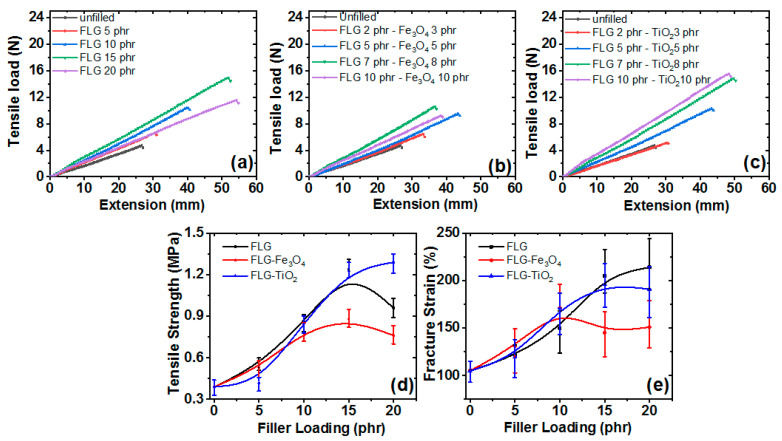
Tensile properties; (**a**) stress-strain plots of RTV-SR/FLG, (**b**) stress-strain plots of RTV-SR/FLG-Fe_3_O_4_, (**c**) stress-strain plots of RTV-SR/FLG-TiO_2_, (**d**) Tensile modulus plots, and (**e**) fracture strain plots of the three composites.

**Figure 10:**
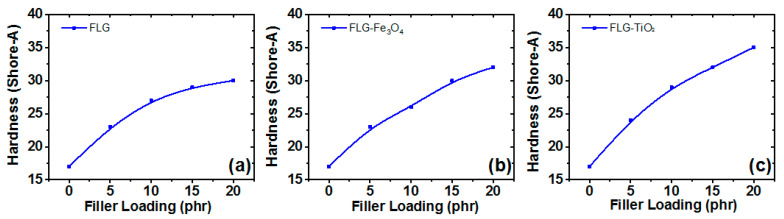
The hardness of the three composites: (**a**) RTV-SR/FLG, (**b**) RTV-SR/FLG-Fe_3_O_4_, and (**c**) RTV-SR/FLG-TiO_2_.

Tensile load and extension were investigated for RTV-SR/FLG (Figure 9a), RTV-SR/FLG-Fe_3_O_4_ (Figure 9b), and RTV-SR/FLG-TiO_2_ (Figure 9c). Tensile loads increased upon increasing filler content. For example, tensile load at a filler content of 5 phr was 6.6 N for RTV-SR/FLG, 6.4 N for RTV-SR/FLG-Fe_3_O_4_, and 5.1 N for RTV-SR/FLG-TiO_2_, while 15 phr increased it to 14.9 N for RTV-SR/FLG, 10.5 N for RTV-SR/FLG-Fe_3_O_4_, and 14.8 N for RTV-SR/FLG-TiO_2_. These increases in tensile load may have been due to the lubricating effect of FLG with increasing filler content in RTV-SR, efficient load-transfer from polymer chains to filler particles, or effective filler networking. Nonetheless, composite hardness increased on increasing filler content (Figure 10).

Tensile strengths (Figure 9d) and fracture strains (Figure 9e) increased upon increasing filler content up to 15 phr and then decreased, except for RTV-SR/FLG-TiO_2_. These results agreed with our compression results in Figure 8. Reductions in the tensile strengths of RTV-SR/FLG and RTV-SR/FLG-Fe_3_O_4_ after 15 phr may have been caused by filler aggregation (see Figure 4) at higher filler contents [58], and we suspected the higher tensile strength of RTV-SR/FLG-TiO_2_ at 20 phr was due to synergism between FLG and TiO_2_ [24,29].

### 3.8. Hardness of the Rubber Composites

Reinforcement by nanofillers such as FLG or hybrid filler (RTV-SR/FLG-TiO_2_ or RTV-SR/FLG-Fe_3_O_4_) played an important role in affecting mechanical, electrical, hardness and viscoelastic properties of the rubber composites. Among these properties, hardness was an important property which improved with increasing filler loading in the rubber matrix. Hardness, especially micro-hardness, was important to determine because high micro-hardness is good enough to break water films on a rubber surface. However, hardness is affected by the high elasticity of the rubber matrix. In order to investigate how soft rubber produced scratches on a glass surface, the hardness of the fillers was considered [59].

Shore A hardness of composites increased with filler content (refer to Figure 10a–c). The variation in hardness between the samples was clear, measured and almost linear as described in Figure 10. The almost linear increases in the hardness of the composites were witnessed with increasing filler content. For example, at a filler loading of 5 phr, Shore A hardness of RTV-SR/FLG, RTV-SR/FLG-Fe_3_O_4_, and RTV-SR/FLG-TiO_2_ were 23, 23, and 24, respectively, and at 20 phr these increased to 30, 32, and 35, respectively, which indicated the fillers had reinforcing and stiffening effects. [9] Furthermore, the hardness of RTV-SR/FLG-TiO_2_ was greater than those of RTV-SR/FLG and RTV-SR/FLG-Fe_3_O_4_ at all filler loadings, which we attributed to the improved polymer-filler interactions and synergistic effects among fillers. The pattern of the results of hardness, especially FLG, was in agreement with ElFaham et al. for graphite-based rubber composites [60]. Rubber composite with a Shore A hardness of 65 or less is rather soft compared to glass [59]. It is possible for composites with stiff fillers to scratch a glass surface depending upon their respective hardness [59].

### 3.9. Actuation Measurements of the Rubber Composites

The RTV-SR can be frequently used as an elastomer slab and electrode in actuation due to its higher dielectric properties. In addition to this, RTV-SR has several benefits when used as an elastomer slab, such as easy processing, lower stiffness, easy to vulcanize, and high chemical resistivity. The actuation displacement can be significantly affected by the thickness of the elastomer slab and many other parameters [61].

**Figure 11:**
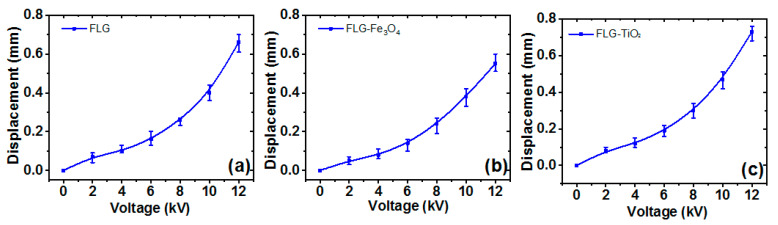
Actuation displacements of: (**a**) RTV-SR/FLG, (**b**) RTV-SR/FLG-Fe_3_O_4_, and (**c**) RTV-SR/FLG-TiO_2_.

Actuation displacements of electrodes prepared using rubber composites with 15 phr of filler are presented in Figure 11. Actuation displacements and voltages for RTV-SR/FLG, RTV-SR/FLG-Fe_3_O_4_, and RTV-SR/FLG-TiO_2_ are shown in Figure 11a–c, respectively. The actuation displacements increased upon increasing input voltage. For example, actuation displacement at 2 kV was 0.07 mm for RTV-SR/FLG, 0.05 mm for RTV-SR/FLG-Fe_3_O_4_, and 0.08 mm for RTV-SR/FLG-TiO_2_. At 12 kV these values increased to 0.66, 0.54, and 0.73 mm, respectively. Notably, actuation displacements were highest for RTV-SR/FLG-TiO_2_ at all voltages. In actuation, the stiffness and thickness of the elastomer slab is known to affect the actuation displacement significantly. Moreover, the limiting factors that further affect the actuation performance are electrical conductivity of the electrode, strain amplitude, and nature of the material used in electrode. The actuation performance further depends on the aspects of the flexibility of elastomeric materials that cause slippage at interfacial areas or the formation of cracks at the electrode under stain. The actuation displacement can be further improved if the electrode made of RTV-SR is replaced with conductive polymer such as Nafion in the electrode material [62].

### 3.10. Magnetic Sensing Ability of the Composites

When a viscoelastic rubber composite is deformed it generates internal strains, which relax with time [63]. Magnetic fields influence this stress-relaxation when a magnetic filler is included in a composite [64,65], and magnetic fillers exhibit anisotropic behaviour in the presence of a magnetic field and isotropic behaviour in the absence of a magnetic field. Thus, magnetic filler containing composites exhibited magnetic sensitivity.

**Figure 12:**
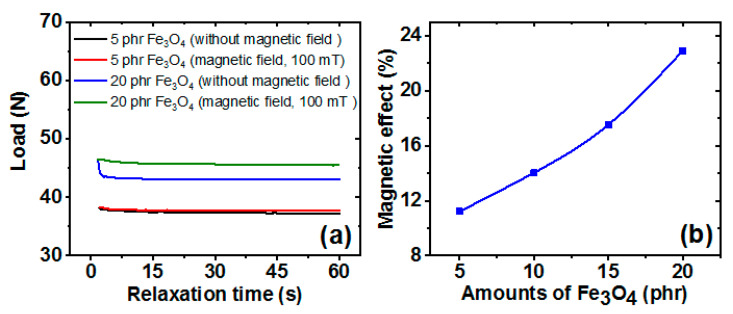
Effect of a magnetic field on stress-relaxation of RTV-SR/FLG-Fe_3_O_4_; (**a**) typical stress-relaxation curves, and (**b**) the effect of magnetic effects on stress-relaxation of RTV-SR/FLG-Fe_3_O_4_ containing different Fe_3_O_4_ loadings.

In this study, the effect of the magnetic effect on stress-relaxation was studied at different Fe_3_O_4_ loadings. Figure 12a shows some typical stress-relaxation curves obtained in the presence and absence of a magnetic field. As shown by the Figure, the stress-relaxation rate decreased when a magnetic field was applied parallel to the deformation axis. Figure 12b shows that the magnetic effect on stress-relaxation values increased with filler loading. Magnetic fields increase filler-filler interactions, and thus, increase magnetic moments of magnetic fillers [66]. It is believed that increasing the Fe_3_O_4_ concentration in the SR composite increased the number of magnetic dipoles, and thus, increased the effect of magnetism on stress-relaxation.

## 4. Conclusions

In the present work, FLG, Fe_3_O_4_, and TiO_2_ nanofillers were used to prepare composites in an RTV-SR polymer matrix. The effects of these nanofillers on mechanical properties, hardness, actuation and magnetic sensor properties were investigated. The functional groups of nanofillers, RTV-SR, and composites were investigated by FTIR and by measuring binding energies by XPS. Tensile testing showed that load increased with increasing displacement and tensile strength increased with filler loading up to 15 phr for RTV-SR/FLG and RTV-SR/FLG-Fe_3_O_4_ hybrid and up to 20 phr for RTV-SR/FLG-TiO_2_. Actuation displacements increased with applied voltage. At 12 kV, actuation displacements were 0.66 mm for RTV-SR/FLG, 0.54 mm for RTV-SR/FLG-Fe_3_O_4_, and 0.73 mm for RTV-SR/FLG-TiO_2_. Stress-relaxation measurements in magnetic fields showed that stress-relaxation rates decreased when magnetic fields were applied parallel to the deformation axis and increased with Fe_3_O_4_ loading.

This study has addressed the use of RTV-SR containing FLG with TiO_2_ or Fe_3_O_4_ for actuators or magnetic sensors. We studied the effects of fillers on mechanical stiffness, actuation and magnetic sensor properties. The target application of this work is soft materials for actuation and MREs. A composite Shore A hardness of below 65 is considered soft and readily available for soft material applications such as flexible devices, actuators, etc. The Fe_3_O_4_ filler used in hybrid with FLG shows a promising magnetic effect at lower filler loadings for use as a magnetic sensor.

## Figures and Tables

**Table 1:** The loading amounts of GNPs in the RTV-SR nanocomposites.

Formulation	RTV-SR (phr)	Fillers (phr)					Hardener (phr)
RTV-SR/FLG	100	3	5	10	15	20	2
RTV-SR/FLG-Fe_3_O_4_	100	3	5	10	15	20	2
RTV-SR/FLG-TiO_2_	100	3	5	10	15	20	2

## Data Availability

Not applicable.

## Supplementary Materials

The following are available online at https://www.mdpi.com/article/10.3390/polym13101550/s1, Figure S1: SEM micrographs at different resolutions at a filler loading of 20 phr: (a,b) unfilled RTV-SR matrix, (c,d) RTV-SR/FLG, (e,f) RTV-SR/FLG-Fe_3_O_4_, and (g,h) RTV-SR/FLG-TiO_2_.
