# Supplementary material for: Properties of Silicone Rubber-Based Composites Reinforced with Few-Layer Graphene and Iron Oxide or Titanium Dioxide
Source: Polymers (Basel). 2021 May 12;13(10):1550. doi: 10.3390/polym13101550 (PMC8151798; doi:10.3390/polym13101550)
Supplement: Supplementary file 1 [file polymers-13-01550-s001.zip › polymers-1223742-supplementary.pdf]

Supporting Information

Properties of silicone rubber-based composites reinforced with few layer graphene and iron oxide or titanium di oxide

Vineet Kumar[a,#], Anuj Kumar[b,#], Min-Seok Song[a], Sung Soo Han[b], Dong Joo Lee[a],

Sang-Shin Park [a]*

[a]*School of Mechanical Engineering, Yeungnam University, 280 Daehak-ro, Gyeongsan 38541, South Korea*

[b]*School of Chemical Engineering, Yeungnam University, 280 Daehak-ro, Gyeongsan 38541, South Korea*

[#]equal contribution

*corresponding author - pss@ynu.ac.kr (SS Park)

Properties of filled RTV-SR composites

Filler dispersions

Filler dispersions in RTV-SR composites at a loading of 20 phr were assessed by SEM (Figure S1) and by elemental mapping. Figure S1 shows that filler particles were uniformly dispersed in the RTV-SR matrix. In high-resolution SEM images, filler particles are indicated by arrows.

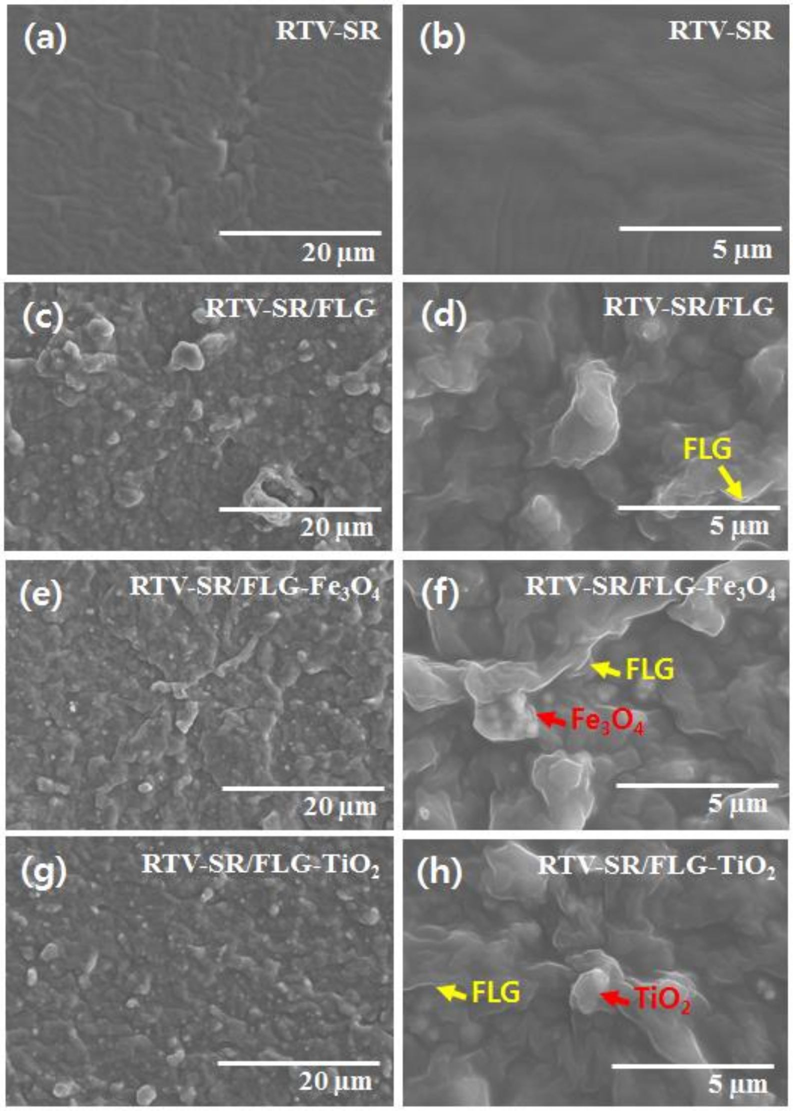

Figure S1. SEM micrographs at different resolutions at a filler loading of 20 phr: (a,b) unfilled RTV-SR matrix, (c,d) RTV-SR/FLG, (e,f) RTV-SR/FLG-Fe_3O_4, and (g,h) RTV-SR/FLG-TiO_2
